# Characterization of the Th Profile of the Bovine Endometrium during the Oestrous Cycle and Early Pregnancy

**DOI:** 10.1371/journal.pone.0075571

**Published:** 2013-10-25

**Authors:** Lilian J. Oliveira, Nadéra Mansourri-Attia, Alan G. Fahey, John Browne, Niamh Forde, James F. Roche, Patrick Lonergan, Trudee Fair

**Affiliations:** 1 Faculty of Food Engineering and Animal Sciences, University of São Paulo, São Paulo, Brazil; 2 School of Agriculture and Food Science, University College Dublin, Dublin, Ireland; Queen's University, Canada

## Abstract

Despite extensive research in the area of cow fertility, the extent to which the maternal immune system is modulated during pregnancy in cattle remains unclear. Therefore, the objective of the current study was to characterize the presence and response profile of B, T-helper (LTh), T- cytotoxic (LTc), gamma delta-T (γδT) and natural killer (NK) lymphocytes in terms of cell number, distribution and cytokine expression in bovine endometrial tissue to pregnancy. Endometrial tissue samples were collected from beef heifers on Days 5, 7, 13 and 16 of the estrous cycle or pregnancy. Samples were analysed by immunofluorescence to identify the presence and abundance of B-B7 (B-cells), CD4 (LTh), CD8 (LTc), γδT cell receptor (TCR) and CD335/NKp46 (NK cells) -positive immune cells. Quantitative real time PCR (QPCR) was carried out to analyse mRNA relative abundance of FOXP3 (a marker of regulatory T (Treg) cells) and a panel of immune factors, including MHC-I, LIF, Interleukins 1, 2, 6, 8, 10, 11,12A, IFNa and IFNG. Results indicate that B-B7+ cells are quite populous in bovine endometrial tissue, CD4+ and CD8+ -cells are present in moderate numbers and γδTCR+ and CD335+ cells are present in low numbers. Pregnancy affected the total number and distribution pattern of the NK cell population, with the most significant variation observed on Day 16 of pregnancy. Neither B lymphocytes nor T lymphocyte subsets were regulated temporally during the oestrous cycle or by pregnancy prior to implantation. mRNA transcript abundance of the immune factors LIF, IL1b, IL8 and IL12A, IFNa and IFNG, expression was regulated temporally during the estrous cycle and LIF, IL1b, IL-10, IL11, IL12A were also temporally regulated during pregnancy. In conclusion, the endometrial immune profile of the oestrous cycle favours a Th2 environment in anticipation of pregnancy and the presence of an embryo acts to fine tune this environment.

## Introduction

Successful pregnancy depends on the precise modulation of maternal immune resources in order to enable the mother to eliminate pathogenic agents if infection occurs, while at the same time providing a receptive and embryotrophic environment for the development of the semi-allogenic conceptus. Studies in mice and humans have implicated a switch from a cell-mediated Th1 immune response to a humoral Th2 immune response in this process [1,2]. Recently the simplicity of this paradigm has been questioned, as it does not account for the implantation period when Th1-type cytokines have been reported to be highly expressed by the endometrium [3,4]. In the cow, functional analysis of microarray data comparing bovine endometrial tissue from pregnant and cyclic heifers have consistently identified the over-population of immune response pathways and processes with differentially expressed transcripts [5-8]. These transcripts may be expressed by endometrial cells and/or by immune cells which are resident or recruited to the endometrium. Previous profiles of the bovine endometrial immune cell repertoire indicate the residence of professional antigen presenting cells (APCs) and CD4+ and CD8+-T cells [9-12]. To-date, there is no information regarding the presence of NK, γδT or Treg cell populations in bovine endometrial tissue. This is in stark contrast to the wealth of information available from studies in mice and humans, where these particular cell types appear to play pivotal roles during implantation (see 13, for review). Furthermore, the activity or cytotoxicity of these cell types appear to be regulated via their interaction with major histocompatability complex (MHC) class I antigens; for example, the non classical Human Leukocyte Antigen (HLA)-G modulates the cytotoxicity of T lymphocytes against the trophoblast [14] and together with non classical HLA-E and classical HLA-C regulates cytokine production and cell lysis by uterine NK cells [15,16] [17,18]. Studies in cattle have identified enhanced expression of classical *MHC-I* transcripts in endometrial tissue [6,19-21] from Day 18 of pregnancy, while non-classical *MHC-I* transcripts have been detected in early cleavage stage bovine embryos [22] and in first and second trimester and term trophoblast tissues [23-25]. Furthermore, studies in our laboratory indicate that the expression of *MHC* class I mRNA by bovine embryos is both transcript- and embryo stage-specific [22] and can be regulated by a number of cytokines including IFNG, IL-4 and LIF [26,27]. Thus, although there is considerably less information relating to the mechanisms modulating the maternal immune response to the semi-allogenic conceptus in cattle, and although implantation and placentation in cattle is quite superficial in contrast to the invasive nature of these events in human, the large body of descriptive data from studies in bovine indicate that the maternal immune response is regulated during early pregnancy in this species. The aim of this study was to characterize the temporal profile of endometrial lymphocyte populations and the regulation of expression of relevant cytokines and MHC class I transcripts during the oestrous cycle and early pregnancy in cattle, focusing on key developmental checkpoints from embryonic genome activation to maternal recognition of pregnancy (MRP). 

## Material and Methods

All experiments involving animals were performed in accordance with the Department of Health and Children, Ireland, as promulgated by the Cruelty to Animals Act (Ireland 1876) and the European Community Directive 86/609/EC. All procedures were sanctioned by the University College Dublin, Ireland, Animals Research Ethics Committee.

### Animal model and tissue collection

#### Expression of immune-related genes in bovine cyclic and pregnant endometrial tissue from Day 5 to Day 16

The experimental design was implemented as previously described [8]. Briefly, cross-bred beef heifers were synchronized to estrus and assigned to either a cyclic group or were artificially inseminated with fertile bull semen from a single proven sire at 12 h after onset of estrus to generate a pregnant group. Animals were slaughtered on Day 5, Day 7, Day 13 and Day 16 following estrus, corresponding to the end of embryonic genome activation, blastocyst formation, initiation of conceptus elongation and maternal recognition of pregnancy, respectively. The uterus was flushed with 20 ml of phosphate-buffered saline (PBS) supplemented with 5% fetal calf serum. Whole uterine cross sections were obtained from the mid-region of the horn ipsilateral to the corpus luteum carrying an intact conceptus at the expected stage of development were used for analysis in the pregnant group (n = 5 heifers per time-point). Similarly, endometrial samples from the mid-region of the ipsilateral horn (n = 5 heifers per time-point) of cyclic heifers were analysed. 

#### Tissue processing for immunofluorescence

Sections of the ipsilateral uterine horn were fixed in 4% paraformaldehyde, then washed in PBS followed by 15% and 18% sucrose solutions. Sections were then immersed in Tissue Tek (Sakura Finetek, Dublin, Ireland) and frozen in liquid nitrogen vapour.

### Immunofluorescent analysis of endometrial lymphocyte populations

Immunofluorescent analysis of bovine endometrial tissue samples, collected on Day 5, 7, 13 and 16 of the estrous cycle and pregnancy, was performed on 4 µm cryo-sections. The sections were fixed in ice-cold acetone for 10 min and air dried for 1 h. Following a rehydration step with 0.05 mM Tris-buffered saline (TBS; pH 7.7), sections were incubated with blocking buffer (TBS supplemented with 10% (v/v) goat serum) for 1 h. All primary antibodies except for mouse anti CD335/NKp46, which was a kind gift from T.Connelly [28], were purchased from VRMD (Pullman, WA 99163, U.S.A.) Single colour immunofluorescence was performed by incubation at 4°C with mouse anti-bovine B-B7 (GB25A, ascites, 1 mg/ml) for B lymphocytes, mouse anti-bovine TcR1-N24 (clone GB21A, ascites, 1 mg⁄mL) for γδT lymphocytes and mouse anti-ovine CD335/NKp46 (clone Gr13.1; ascites, 1 mg⁄ml) for Natural Killer cells [28]. Two-colour immunofluorescence labelling was carried out for CD4^+^ and Cd8^+^ T lymphocytes by concomitant incubation of sections with primary antibodies: mouse anti-bovine CD4 (clone IL-A11, ascites, 1 mg ⁄ml) and mouse anti-bovine CD8 (clone BAQ11A, ascites, 1 mg/mL) for T lymphocytes. Negative controls were labelled with a relevant isotype control (10 mg/ml) at the same concentration as the primary antibody. The sections were then washed 3 times in TBS and incubated with secondary antibodies diluted 1:800 [either Alex Fluor 488 goat anti-mouse IgG (H&L)(cat #A-1109) or Alex Fluor 594 goat anti-mouse IgG (H&L) (2 mg/mL; cat #A-11032, Invitrogen Eugene, OR, USA] for 30 min at room temperature. Following repeated washing in TBS, the sections were incubated with DAPI (1 mg/ml for 15 min) for DNA labelling, washed again and cover slips were mounted using Prolong Antifade mounting medium (Invitrogen). The slides were examined using a Zeiss Axioplan 2 epifluorescence microscope (Zeiss, Gottingen, Germany) with Zeiss filters 02 (DAPI filter), 03 (FITC filter) and 15 (rhodamine filter) at 40 X magnification. Digital images were acquired using AxioVision software (Zeiss) and a high-resolution black and white Zeiss AxioCam MRm digital camera. Five random fields within each endometrial region were captured for morphometric analysis: luminal epithelium (LE), shallow stroma (SS), deep stroma (DS) and myometrium (M) and the number of positive cells were counted in each region, within a 2.25 mm^2^ field of view. For the epithelium, squares were chosen for counting so as to ensure that the entire area of the square was within the epithelium. A total cell number was generated and analysed for each sample based on the sum of the average number of cells per field of view in each of the four regions. Therefore the total cell number reflects the number of cells in 9mm^2^. Data were analysed separately for each of the four regions and for the sum of the four regions by least square analysis of variance using the General Linear Models procedure of SAS (SAS Institute Inc., Cary, NC, USA). The model included effects of treatment (pregnant vs. non pregnant) and day after AI.

#### Tissue processing and mRNA expression analysis by quantitative real time PCR 

300 mg strips of endometrium (predominantly intercaruncular tissue) were immersed in 1:5 wt/vol RNAlater and transferred to RNase/DNase-free tubes and stored at −80°C for RNA extraction. Total RNA was extracted from approximately 100 mg strips of intercaruncular endometrium using Trizol reagent (Invitrogen), followed by on-column DNase digestion and RNA clean up using the Qiagen mini kit (Qiagen, Crawley, West Sussex, UK) as per manufacturer’s instructions. Complementary DNA was synthesized from 1 μg of purified total RNA. The mRNA expression profiles of selected candidate genes were analysed by quantitative real-time PCR (qPCR) using the ABI Prism 7500 FAST sequence detection system and Fast SYBR Green Master Mix (Applied Biosystems, Warrington, UK). Primers were designed for each gene of interest (Primer Express Software v2.0, Applied Biosystems) (Table S1 in File S1). The specificity of all primers was confirmed both by melt-curve analysis and by sequencing of the amplified PCR fragments. Primer efficiency was determined using a serial dilution of *Bos taurus* derived cDNA (1:4 dilution series over 7 points). The optimal number of reference targets for this sample set were identified using the geNorm application within the qbase^PLUS^ software package [29] (Biogazelle, Zwijnaarde, Belgium) and confirmed for this study (geNorm V < 0.45). The normalization factor was calculated as the geometric mean of reference targets *ACTB*, *RPL19* and *PPIA*. Calibrated normalised relative quantities (CNRQ) of gene expression for each analysed sample were generated by the qbase^PLUS^ package. All statistical analyses were carried out using the SAS v9.1.3 software package (SAS Institute, Cary, NC, USA). As the data did not approach a normal distribution as determined by the box-cox transformation in the TRANSREG procedure of SAS [30], all values were transformed using a Log^^10^ transformation. CNRQ measurements were analysed using a linear mixed model (PROC MIXED). The statistical model included the effects of treatment (pregnant or cyclic) and time (day of pregnancy or day of estrous cycle), and all possible interactions. These effects and their interactions were included in the model if their P-value was <0.25. All candidate gene CNRQs were included in the initial model as covariates. Acceptable significance levels were declared p<0.05. A summary of the covariates significantly affecting candidate gene expression is presented in Table 1. A Bonferoni adjustment was used to account for multiple comparisons. Gene expression is plotted as log_10_ least-square means ± SEM or summarized as geometric mean fold-changes following back-transformation of least-square means. Pearson correlations were estimated between all genes for pregnant and cyclic cows at days 5,7, 13, and 16 and correlations with p<0.05.

**Table 1:** Summary of covariates significantly affecting candidate gene mRNA expression in endometrial tissue.

**Gene ID**	**Day**	**Status**	Status*day	**Transcript**
*BOLA (MHC-I)*		0.02	0.26	IL1B, IL8, NC1,NC2,NC3, PTX3
*FOXP3*	0.058		0.08	MCP2, NC3, NC4
*IFNA*	0.0001		0.29	CSF1, IL6
*IFNG*	0.0356			IL1B, IL8, MCP2, ISG15
*LIF*	0.0001	0.23	0.27	
*IL1A*			0.8	IL15
*IL1B*	0.0001		0.59	IL8, LIF, MCP2
*IL2*	0.2	0.06	0.16	
*IL6*	0.036	0.13	0.3	LIF
*IL8*	0.0005		0.85	BOV7/11, FOXP3, IL1B
*IL10*	0.0001			IFNA, IL8
*1L11*	0.0001	0.024	0.0075	NC4
*IL12A*	0.04	0.13	0.004	BOV7/11, IL15, 1L11, 1L6

## Results

### Characterization of bovine endometrial lymphocyte population

Our findings indicate that the bovine endometrium plays host to B-B7^+^ lymphocytes, CD4^+^, CD8^+^, γδT^+^ and possibly T-reg (see QPCR *FOXP3* results below) subsets of T-lymphocytes and NK -cells. A total cell number was generated from the sum of the average number of cells per field of view (2.25 mm^2^) in each of the four regions (LE, SS, DS and M), so that the total cell number reflects the number of cells in 9mm^2^. Total cell numbers were highest, 100-200 cells for B-lymphocytes which often presented in aggregates, moderate (20-140 cells) for T-lymphocytes and NK cells and quite low for γδT –cells (<16) in the bovine endometrium.

### Lymphocyte profile during the estrous cycle and early pregnancy

The populations of γδTCR^+^, CD4^+^, CD4^+^ and B-B7^+^ -cells did not appear to be regulated by the day of the estrous cycle. However, there was a large variation in the numbers of γδT and CD4^+^-cells between samples/animals. There was a progressive increase in total NK cell number during the luteal phase, culminating in a dramatic rise on Day 16 of the cycle (p<0.005). There was no apparent response to the presence of an embryo in the pregnant endometrium by any of the T-cell subsets. However, the presence of an elongating embryo appeared to suppress the expansion of the small NK cell population as numbers were maintained at a constant level in contrast to their dramatic expansion at Day 16 of the estrous cycle.

### Lymphocyte localization in the endometrium

In general, the highest concentrations of lymphocytes were localized in the stromal tissue irrespective of pregnancy status or day of sampling. The luminal epithelium was primarily devoid of CD4^+^ and CD8^+^-T cells and B-B7^+^ -B cells, but hosted a very sparse population of γδT and NK -cells. 

#### B-B7^+^

**Figure 1:**
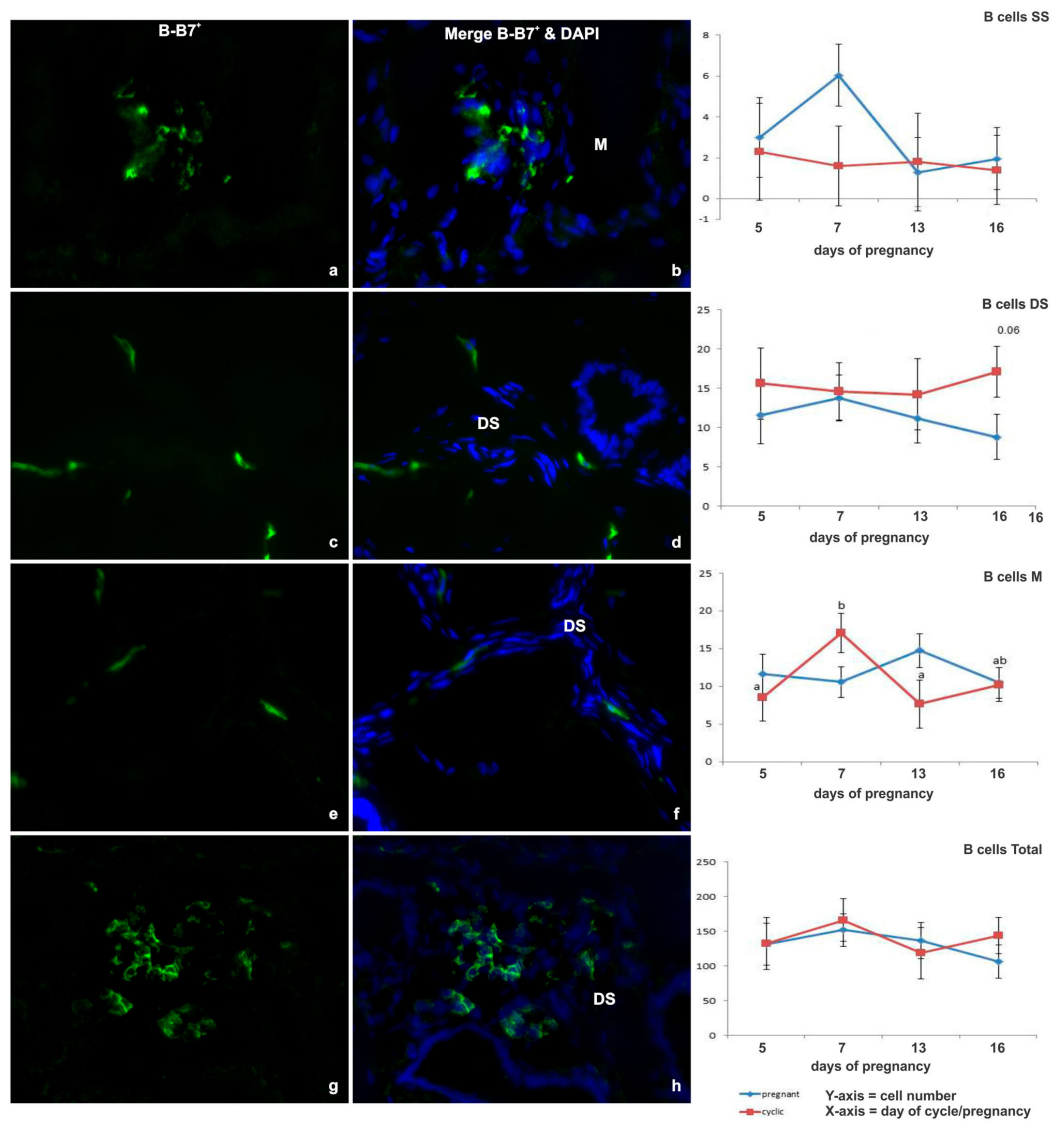
Immunofluorescent labeling of endometrial tissue for B cells. (a-h) Representative images of immunofluorescent B cells^+^ (B-B7) labeling (green) in cells (nuclei labeled with DAPI [blue]), in the endometrial myometrium (M) and deep stroma (DS) of cyclic (C) heifers on Day 7 & 16 and in the DS from pregnant (P) heifers on Day 7 & 16 (original magnification X40). (i-l) Line graphs detailing the total number of B cells in cyclic (red line) and pregnant (blue line) endometrial tissue based on the sum of immunofluorescent labeled cells per 2.25 mm^2^ field of view, in all areas (total B-B7^+^ cells) and specifically in the shallow stroma (SS), DS and M. Values plotted are least squared means and standard errors across 5 replicate animals per timepoint and per status.

The highest concentrations of cells were located in the deep stroma or myometrium. The cells localized in the myometrium were exclusively located around blood vessels, whereas the cells localized to the stroma were frequently found in small aggregates (Figure 1a-h). Neither the distribution pattern, nor the total number of B-B7^+^ lymphocytes in the endometrium were regulated temporally, or by pregnancy (Figure 1i-l).

#### CD4^+^ and CD8^+^

**Figure 2:**
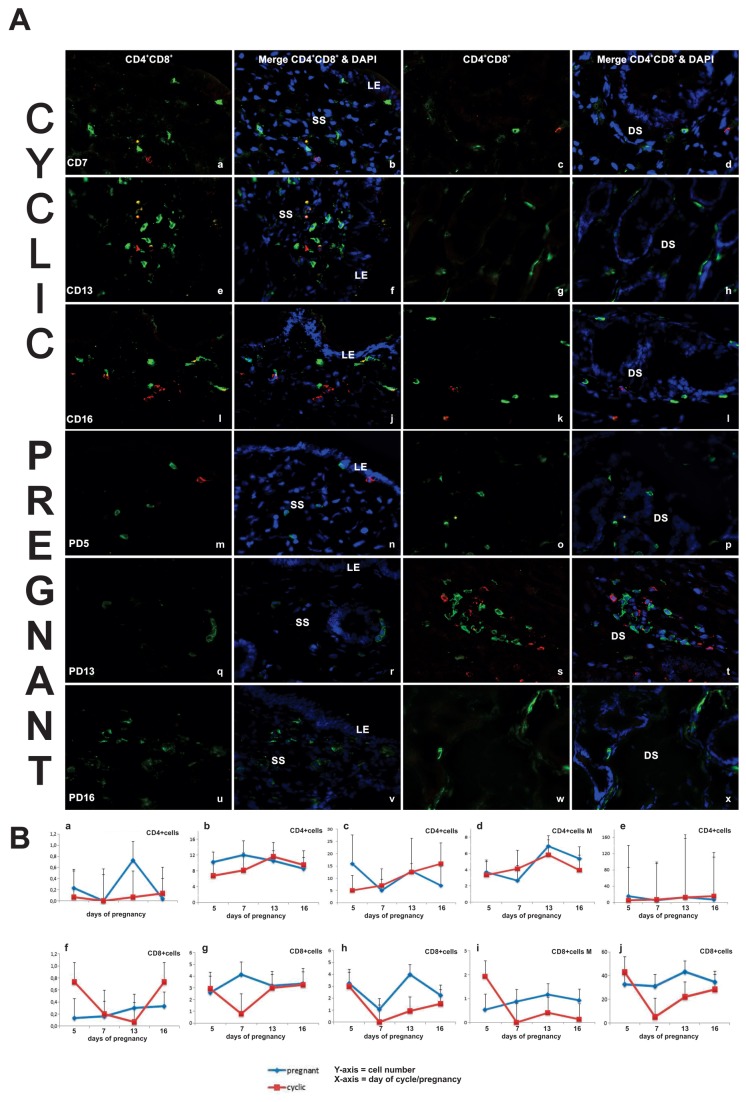
A) Immunofluorescent labeling of endometrial tissue for CD4^+^ and CD8+ T cells. (a-j) Representative images of immunofluorescent CD4^+^ cells labeling (green) and CD8^+^ cells labeling (red) and nuclei labeled with DAPI [blue]), in the endometrial shallow stroma (SS) ; deep stroma (DS) and myometrium (M) in cyclic at day 7 (a-d) ; 13 (e-h) and 16 (i-l) of estrus cycle and at day 5 (m-p) ; 13 (q-t) and 16 (u-x) of pregnancy. (original magnification ×40). B) Line graphs detailing the total number of endometrial CD4+  and CD8+ cells. CD4+ (a-e) and CD8+ (f-j) -cell numbers in cyclic (red line) and pregnant (blue line) endometrial tissue are based on the sum of immunofluorescent labeled cells per 2.25 mm^2^ field of view, in all areas (total CD4+ or CD8+) and specifically in the luminal epithelium (LE), SS, DS and M. Values plotted are least squared means and standard errors across 5 replicate animals per timepoint and per status.

**Figure 3:**
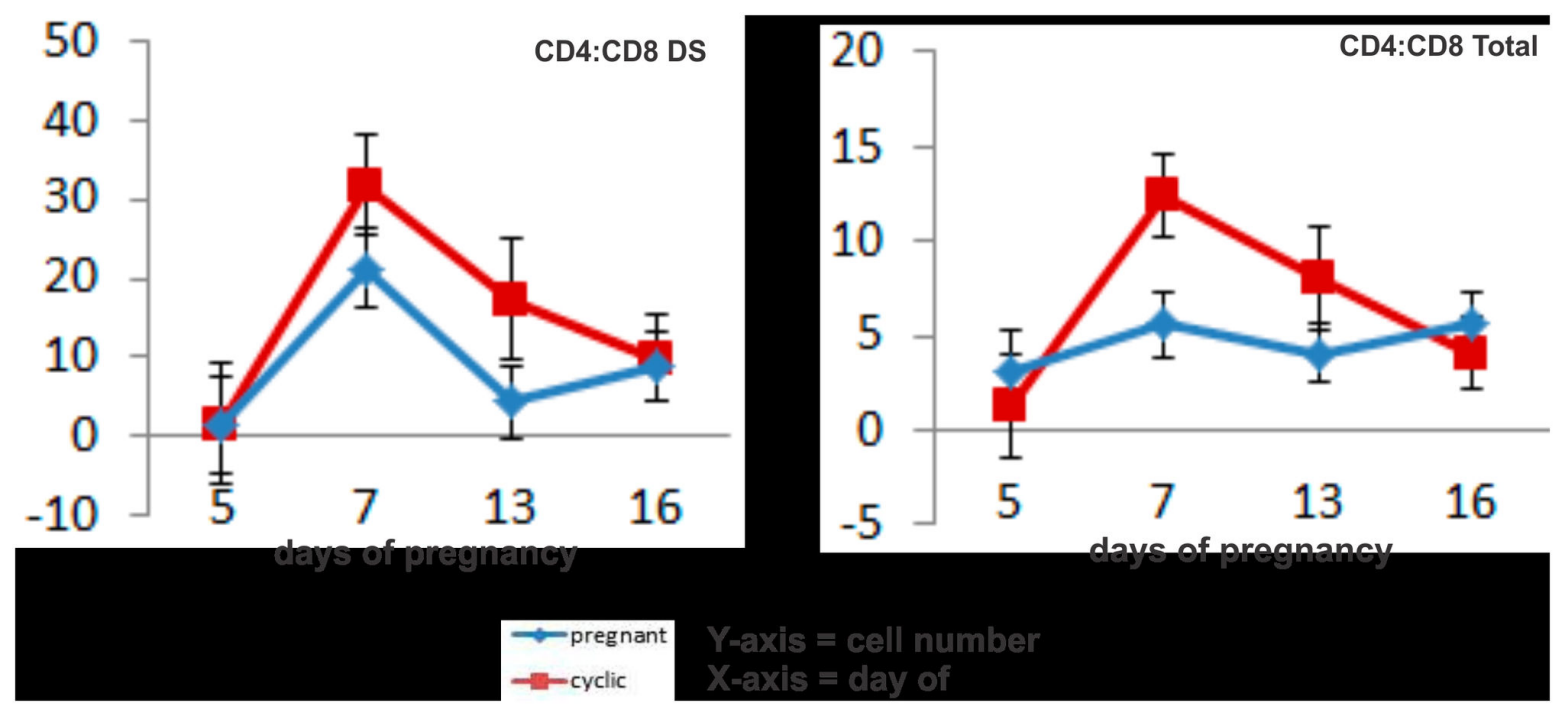
Line graphs detailing the CD4 :CD8 ratio in cyclic (red line) and pregnant (blue line) endometrial tissue. Values plotted are the ratio of least squared means and standard errors of immunofluorescent labeled cells per 2.25 mm^2^ field of view, specifically in the deep stroma (DS) and the sum of immunofluorescent labeled cells per 2.25 mm^2^ field of view, in all regions (total cells) across 5 replicate animals per timepoint and per status,.

The majority of CD4^+^ and CD8^+^ cells were located in the shallow areas of the endometrial stroma; just a few positive cells were observed within the luminal epithelium (Figure 2A a-d and m-p). CD4^+^ cells in the glandular stroma were mostly peri-glandular, but sometimes intraepithelial in location (Figure 2A e-h and q-t). CD4^+^ cells were present in very small numbers in the myometrium, primarily in the perivascular region (Figure 2A i-j and u-x). CD8^+^ cells were concentrated in the shallow stroma and occasionally within the luminal epithelium. The CD4^+^ cells were present in higher numbers compared to CD8^+^ cells. The total number of CD4^+^ and CD8^+^ cells in the endometrium was not regulated temporally or by pregnancy (Figure 2B a-e and f-j). The ratio of CD4^+^ to CD8^+^ -T cells increased significantly in the deep stroma of both cyclic and pregnant tissue on Day 7 compared to Day 5 (p<0.05), but did not differ significantly between pregnant and cyclic tissue samples (Figure 3).

#### γδTCR^+^ cells

**Figure 4:**
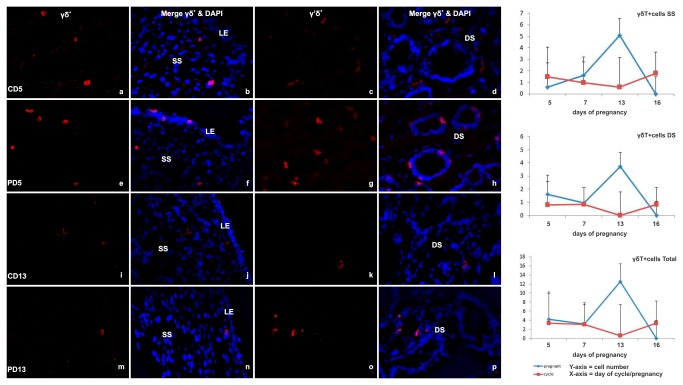
Immunofluorescent labeling of endometrial tissue for γδT-cells. (a-f) Representative images of immunofluorescent γδT cell receptor (WC1) labeling (green) in cells (nuclei labeled with DAPI [blue]) of the endometrial deep stroma (DS) from cyclic (C) Day 5 and pregnant (P) Day 7 and 16 -heifers (original magnification ×40). (g-i) Line graphs detailing the total number of γδT-cells in cyclic (red line) and pregnant (blue line) endometrial tissue based on the sum of immunofluorescent labeled cells per 2.25 mm^2^ field of view, in all areas (total γδT-cells^+^) and specifically in the SS and DS. Values plotted are least squared means and standard errors across 5 replicate animals per timepoint and per status.

The γδT cell population was very sparse in the majority of endometrial samples analysed. These cells were primarily concentrated in the stromal and myometrial layers (Figure 4a-f). There was no difference between cyclic and pregnant animals in terms of numbers and distribution of γδT cells (Figure 4g-i).

#### NKp46 (CD335^+^)

**Figure 5:**
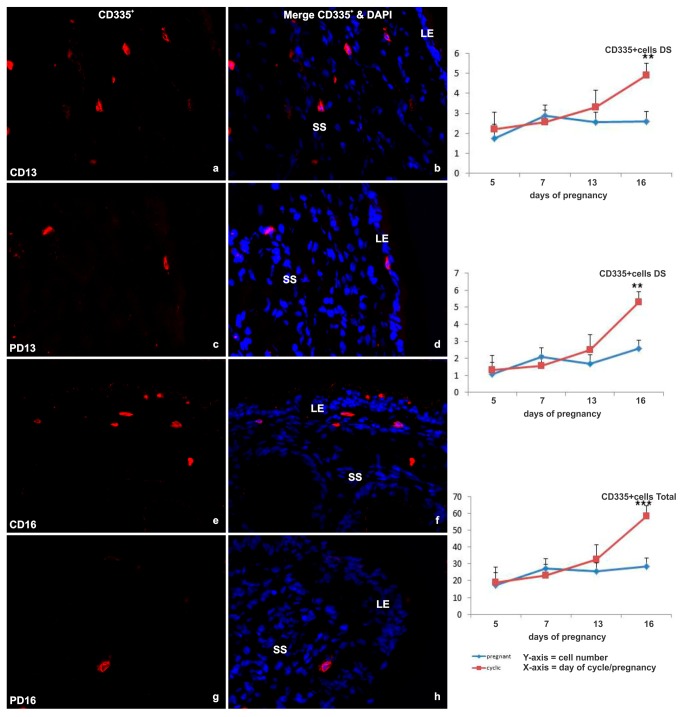
Immunofluorescent labeling of endometrial tissue for NK cells. (a-h) Representative images of immunofluorescent CD335^+^ NK cell receptor labeling (red) in cells (nuclei labeled with DAPI [blue]) of the endometrial SS from cyclic (C) and pregnant (P) heifers on Days 13 and of the deep stroma (DS) from C and P heifers on day 16 (original magnification ×40). B) Line graphs detailing the total number of NK cells in cyclic (red line) and pregnant (blue line) endometrial tissue based on the sum of immunofluorescent labeled cells per 2.25 mm^2^ field of view, in all areas (total CD335^+^) ) and specifically in the luminal epithelium (LE), shallow stroma (SS) and in the deep stroma (DS). Values plotted are least squared means and standard errors across across 5 replicate animals per timepoint and per status.

CD335^+^ NK cells displayed a similar distribution to the lymphocyte populations described above. CD335^+^ cells were present mainly in the shallow stroma underlying the luminal epithelium (Figure 5a-h). Some scattered cells were observed in the glandular stroma and in the myometrium. The total number of NKp46 (CD335^+^) cells remained low until Day 16, when a significant increase was observed in the deep stroma (p<0.001) in the cyclic endometrium (p<0.005). In contrast, there was no increase in NK cells in the endometrium of pregnant animals, such that the total number of CD335^+^ cells was 2-fold lower than in cyclic endometrium (p<0.001), particularly in the superficial stroma (p<0.07) and deep stroma (p<0.02) (Figure 5i-k).

### Characterization of bovine endometrial cytokine and immune factor expression

#### Temporal regulation during the estrous cycle 

**Figure 6:**
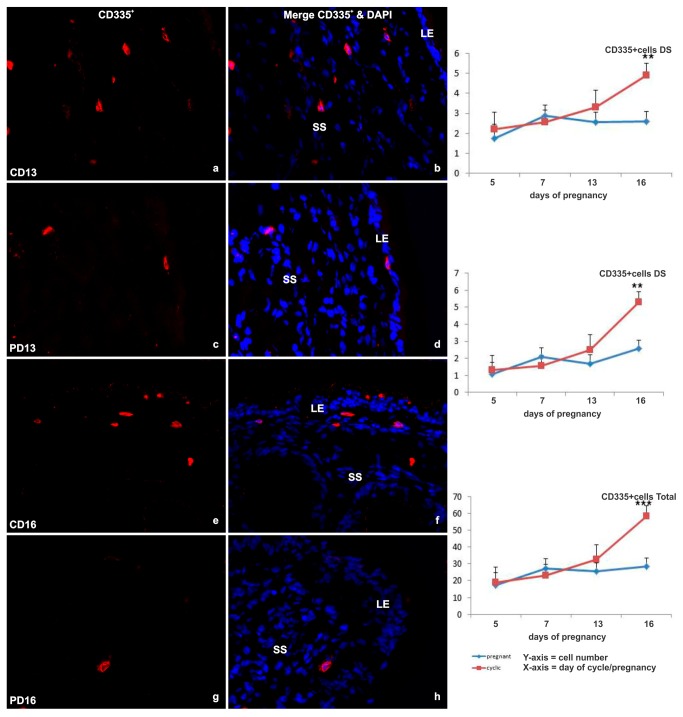
Cytokine mRNA expression profiles. mRNA abundance of selected cytokines in cyclic (red line) and pregnant (blue line) endometrial tissue during the pre- and peri-implantation period. Values plotted are least squared means and standard errors across across 5 replicate animals per timepoint and per status. Asterisks indicate significant difference between pregnant and cyclic tissue at a particular time point: * = *P* < 0.05.

The mRNA expression levels of a panel of immune-related genes (n=13) was profiled in the bovine endometrium on Days 5, 7, 13 and 16. Expression of *IFNA, LIF, IL1B, IL8* and *IL12A* was regulated temporally during the estrous cycle (P<0.05). Briefly, *IFNA* and *IL1B* transcript abundance increased during the early luteal phase but was significantly suppressed in the late luteal phase. In contrast, expression of *IL-12A* and *IFNG* increased from the mid luteal phase to the late luteal phase, while *IL8* expression was highest early in the cycle, declined during the mid luteal phase and increased again at the late luteal phase. *LIF* expression progressively increased through the estrous cycle, plateauing during late luteal phase. The mRNA expression profiles of *MHC-I* transcripts, *FOXP3*, *IL1A, IL2, IL6, IL10* and *IL-11* were not significantly (P>0.05) temporally regulated during the estrus cycle. The data are summarized in Table S2 in File S1 and illustrated in Figure 6.

#### Temporal regulation during pregnancy

The mRNA expression profiles of the panel of candidate immune-related genes during early pregnancy was characterized in bovine endometrial tissue from the arrival of the morula stage embryo in to the uterine horn on Day 5 until MRP on Day 16. Briefly, expression of *LIF* and *IL-10* increased up to Day 13, after which expression plateaued, whereas expression of *IL12A* increased steadily until MRP on Day 16. In contrast, *IL-1B* and *IL11* mRNA expression was highest on Day 7 and lowest on Days 13 and 16. The mRNA expression profiles of *MHC-I* transcripts, *FOXP3*, *IFNA*, *IL1A, IL2, IL6*, and *IL-8* were not significantly (P>0.05) temporally regulated during early pregnancy. The data are summarized in Table S3 in File S1 and illustrated in Figure 6.

### Analysis of correlations in gene expression

The expression profiles of a panel of 32 immune factors were analysed to determine which factors were correlated. The results are summarized in Table S4 in File S1 for genes that were temporally regulated during the estrous cycle or pregnancy. In general, there was considerable interaction between factors and there was no clear influence of Th 1 type or Th 2 type cytokines. However, the influence of interferon-stimulated genes, *PTX3*, *RSAD2* and *ISG15* was primarily in effect on Days 13 and 16 of pregnancy, when their expression is highest [31].

## Discussion

The current study presents a detailed description of the principle lymphocytes hosted by the bovine endometrium during the estrous cycle and early pregnancy up to and including maternal recognition of pregnancy. Endometrial expression of key immune-related genes was carried out in parallel and is described from the arrival of the embryo in the uterine horn on Day 5 through blastocyst formation on Day 7, initiation of elongation on Day 13 and MRP on Day 16. The bovine endometrium is host to populations of B- and T-lymphocytes which are primarily located in the stromal tissues. The lymphocyte population is composed primarily of B-cells, T-cells and NK cells in both pregnant and cyclic endometrium. In agreement with earlier findings [10], we noted that B-lymphocytes were widely distributed throughout the endometrium, localizing in the stroma, the luminal and glandular epithelium and in the myometrium. A small number, or absence of CD5^+^ B-cells has been reported previously [9]; however, later results using CD21 as a B-lymphocyte marker showed the presence of B-cells in the myometrium and deep areas of glandular endometrium in the cow [10]. In the current study, there was no difference in the number of B-lymphocytes in the endometrium between pregnant and non-pregnant cycling heifers, irrespective of stage analyzed. The B-lymphocyte population was relatively large compared to the populations of γδT, CD4^+^, CD8^+^ and NK -cells detected; however, their numbers were similar to those reported previously for dendritic cells and macrophages [31]. 

There was great variation among animals with regard to the size of the population of CD4^+^ cells, as has been previously reported [10]. Although the reason behind such high variability is not clear, there is evidence that the stress and/or health status of each animal impacts on their immune cell profile [32]. In contrast to the findings of Cobb and Watson [9], our results did not show any evidence of estrous cycle regulation of total endometrial CD4^+^ or CD8^+^ cell numbers, nor a preponderance of CD8^+^ cells in the luminal epithelium. In fact, both CD4^+^ and CD8^+^ -cells were primarily located in the stromal tissue of endometrium, which agrees with previously published data [10]. 

In the current study we report for the first time, direct evidence of the presence of γδT cells in both pregnant and cyclic bovine endometrium; however, they were very few in number and were present in all strata observed. The only previous evidence of the presence of γδT cells in the bovine endometrium is from a global transcriptome analysis that revealed that the mRNA expression of γδT-receptors was higher in lactating compared to non-lactating cows, regardless of the reproductive status [33]. Furthermore, there was no evidence of their regulation during the estrous cycle. In sheep, the γδT cell population expands during mid- to late-pregnancy when they are believed to play an important role in the control of uterine infection pre and post parturition and may also have an active function in the parturition process [34-36].

 In humans, NK cells constitute up to 70% of the endometrial lymphocyte population during the fertile phase of the menstrual cycle and the perimplantation phase of pregnancy [15]. In the cow, we observed a small population of NK cells in the bovine endometrium which was sparsely distributed across all strata. The total uterine NK (uNK) cell number increased incrementally during the luteal phase, culminating in a dramatic rise on Day 16 (p<0.005) of the estrous cycle. A similar peak in uNK cell numbers in the late luteal phase has been described in humans (review, [37]). These levels are maintained in humans during early pregnancy. However, uNK cell numbers remained static from Days 5 to 16 in the pregnant presence of a conceptus or conceptus-derived signal(s) has a suppressive effect samples of the current study. The lack of expansion in the presence of a conceptus or conceptus-derived signal(s) is supported by in vitro studies which demonstrated anti-proliferative effects of recombinant IFN-τ exposure on immune and uterine cells, particularly leukocytes, which were up to 1000 times more sensitive to IFN-τ than stromal cells [38]. However, recent preliminary data from cattle has indicated an increase in the uNK cell population in Day 17 pregnant endometrial tissue compared to non-pregnant counterparts [39]. The difference in findings between the two studies might be explained by methodology and/or breed differences, as the authors used flow cytometry of digested dairy heifer endometrial tissue to identify uNK cells. Comparing human and mouse uterine NK (uNK) cell data in light of the alteration of the vasculature required to support implantation in both species, has lead to the hypothesis that uterine NK (uNK) cells play a pivotal role in local vascular remodeling and regulation of trophoblast invasion (for review see 4,13). Implantation in cattle is noninvasive and begins from day 19 onwards, with the development of chorionic cotyledons on the trophoblast which fuse with superficial caruncular structures on the endometrial epithelium to form placentomes (see review by Bazer et al., [40]). The formation of these structures requires the increased vascularisation of discrete areas within the endometrium indicating a role for uNK cells during implantation in cattle. Taken together these findings imply IFNT regulation of a complex series of events which promote the elongation of the embryo, thus maximising surface area for nutrition in the absence of trophoblast invasion and immunological protection of the conceptus through the upregulation of antiviral factors [31] while suppressing uNK cell expansion until required.

The presence and regulation of Treg cells was assessed by QPCR analysis of the Treg cell-specific marker Foxp3. In contrast to observations in human and mouse, where Treg cells accumulate in the uterus in anticipation of implantation [41,42], this subset of T cells was neither regulated temporally during the estrous cycle, nor by any stage of early pregnancy in cattle. Thus, it would appear that the expansion of the Treg cell population is not required for implantation, but maybe important during placentation, as the percentage presumed Treg cells (CD4^+^ CD25^+^ T cells) was increased in pregnant cows at Day 33-34 of pregnancy [12]. Similarly, QPCR analysis of *FOXP3* expression in lymphocyte samples collected from post Day 40 pregnant and non-pregnant mares indicated that there was no change in the PBMC Treg population during early equine pregnancy, but increased numbers of FOXP3^+^ CD4^+^ T cells were identified around the endometrial cups compared to peripheral blood [43]. Thus, the expansion of the endometrial Treg cell population in response to pregnancy appears to be tightly regulated in cattle and mares from in terms of timing and localization.

### Regulation of Th-related cytokines

The premise that physiological changes observed in the endometrium from Days 1 to 16 of the bovine estrous cycle occur as default in anticipation of pregnancy [8] is supported by the absence of differences in cytokine transcript abundance between cyclic and pregnant tissue in the current study. Nevertheless, the findings of the current study suggest that in cattle the default profile in anticipation of pregnancy is a trend towards a Th2 environment, as proinflammatory factors such as *IFNA, IL1B, IL8* and *IL11* were lower in the late luteal phase of the cycle and/ or coinciding with MRP, whereas, both *IL10* and *LIF* expression increased steadily. Correlation analysis of immune factor expression indicated that *IFNA* expression was correlated with the expression of the powerful Th2 cell-derived suppressant *IL10* [3], whereas the expression of all other factors were primarily correlated with the expression of macrophage-derived factors such as the *IL12, IL15* and *IL18* trio and *MCP1* and *MCP2*. The trend in TH2 bias appeared to be fine-tuned by pregnancy as temporal down regulation of *IL1A* and *IL11* were more marked in pregnant tissue samples. Furthermore, the ‘correction’ appeared to be at the level of the cytokine environment as the broad lymphocyte population profile is unchanged. In women, both IL1 and IL11 are involved in the local regulation of immune cell invasion and endometrial remodelling during a normal menstral cycle (IL1) [44,45] or early implantation (IL11) [46,47]. It is likely that the temporal downregulation of *IL11* expression observed in the pregnant bovine endometrium is associated with a parallel temporal suppression of expansion of the NK cell population at this stage of early pregnancy in cattle, as experiments in mice have shown that IL11 signaling is required for decidual-specific maturation of NK cells [48]. 

The progressive increase in *LIF* mRNA expression observed in both cyclic and pregnant tissues up to Day 16 is consistent with its pronounced expression in mid- and late- secretory phase human and mouse endometrium (for review see 4,49-52. LIF is regarded as an important factor in both murine and human embryo implantation; it is a key regulator of decidualization [53] and LIF-deficient female mice are infertile due to a failure of implantation [54]. LIF may also act to regulate *MHC-I* expression by developing embryos [27]. Similarly, IL10 is expressed abundantly in the decidual and placental tissues of mice and humans [55] ; however, mouse *IL10* deletion studies have shown that it is not essential for normal pregnancy outcome [56]. Nevertheless, IL10 is a central regulator of the inflammatory response, acting to regulate monocyte and macrophage synthesis of TNFA and other pro-inflammatory cytokines and chemokines in the uterine deciduas [57,58]. Recent data from our group indicates that there is an expansion of monocyte-derived macrophages and dendritic cells by Day 13 of pregnancy in cattle, without an increase in *TNFA* expression [31]. In addition, IL10 has been shown to protect pregnancy from the adverse effects of inflammatory challenge [59]; therefore, upregulation of *IL10* expression in the bovine endometrium may reflect a maternal immunoprotection mechanism, or the induction of an M2 phenotype, by the endometrial macrophages required for endometrial remodeling and or fetal acceptance [57,60]. Consistent with our immunohistochemistry and *FOXP3* QPCR observations that the CD4^+^, CD8^+^, γδTCR^+^ and Foxp3 -T-cell populations are neither regulated temporally up to Day 16 of the estrus cycle, nor prior to MRP of pregnancy, apart from *IL10*, we did not see temporal regulation of factors uniquely associated with T-lymphocytes. In contrast, we have previously reported extensive regulation and correlations in expression of macrophage and dendritic cell derived factors [31]. 

In conclusion, the findings of this study demonstrate for the first time, the presence of NK and γδT -cells in the bovine endometrium. Here we report that the maternal endometrial lymphocyte population is not modified up to Day 16 of the estrus cycle or pregnancy. However, the lymphocyte activation status appears to be tightly regulated as proinflammatory factors such as *IFNA, IL1B, IL8* and *IL11* are lower in transcript abundance in the late luteal phase of the estrous cycle and/ or coinciding with MRP; in contrast, the expression of both *IL10* and *LIF* increased steadily, suggesting that in cattle, there is a trend in cytokine expression a trend towards a Th2 environment in anticipation of pregnancy. 

## Supporting Information

File S1 **Trial Protocol.**
(DOCX)
