# Correction: Characterization of the Th Profile of the Bovine Endometrium during the Oestrous Cycle and Early Pregnancy

**DOI:** 10.1371/annotation/93d63399-0e71-4a25-a45c-311910ee6da5

**Published:** 2014-01-16

**Authors:** Lilian J. Oliveira, Nadéra Mansourri-Attia, Alan G. Fahey, John Browne, Niamh Forde, James F. Roche, Patrick Lonergan, Trudee Fair

A term was incorrectly capitalized in the fifth sentence of the Abstract. The fifth sentence of the Abstract should read: "Samples were analysed by immunofluorescence to identify the presence and abundance of B-B7 (B-cells), CD4 (LTh), CD8 (LTc), γδT cell receptor (TCR) and CD335/NKp46 (NK cells) -positive immune cells. Quantitative real time PCR (QPCR) was carried out to analyse mRNA relative abundance of FOXP3 (a marker of regulatory T (Treg) cells) and a panel of immune factors, including MHC-I, LIF, Interleukins 1, 2, 6, 8, 10, 11,12A, IFNA and IFNG."

In addition, Figures 3 and 6 are incorrect, please view the correct Figures 3 and 6 below. 

Figure 3: 

**Figure:**
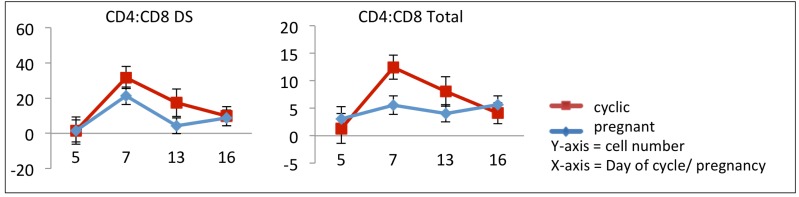


f

Figure 6: 

**Figure:**
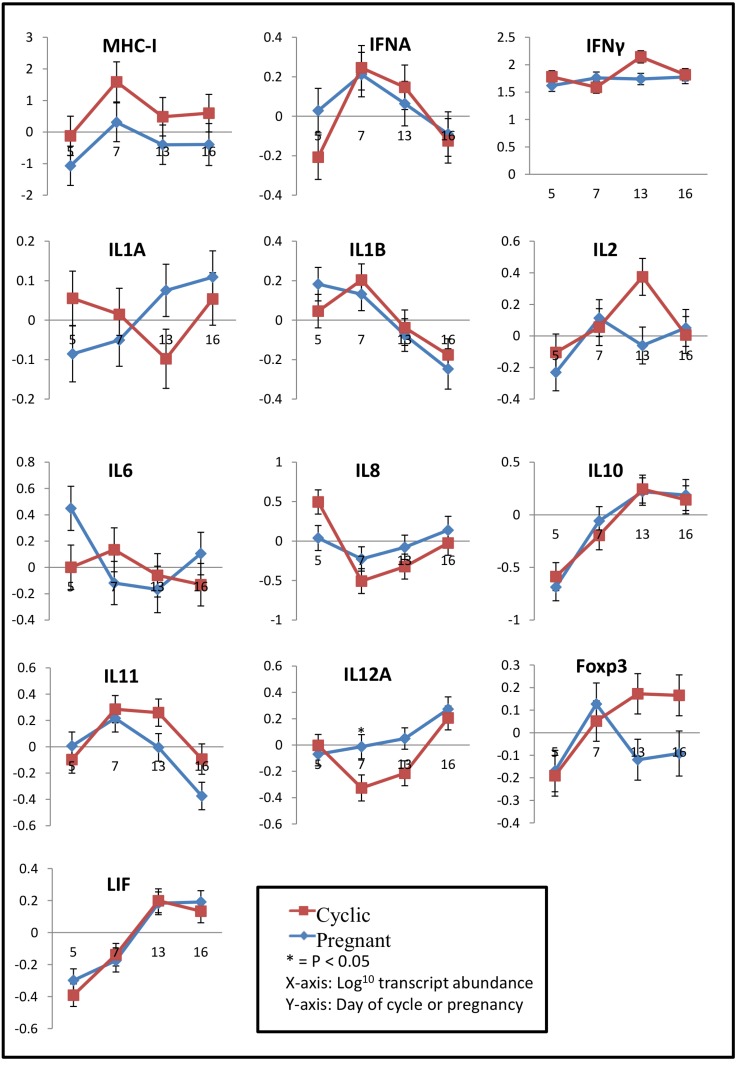


f

In addition, there was an error in the first sentence of the legend of Figure 4. The first sentence of the legend of Figure 4 should read: "(a-f) Representative images of immunofluorescent γδT cell receptor (WC1) labeling (red) in cells (nuclei labeled with DAPI [blue]) of the endometrial deep stroma (DS) from cyclic (C) Day 5 and pregnant (P) Day 7 and 16 -heifers (original magnification ×40)."

