# Correction: Characterization of the Th Profile of the Bovine Endometrium during the Oestrous Cycle and Early Pregnancy

**DOI:** 10.1371/journal.pone.0114116

**Published:** 2014-11-17

**Authors:** The PLOS ONE Staff

The second author’s name is spelled incorrectly. The correct name is: Nadéra Mansouri-Attia. The correct citation is: Oliveira LJ, Mansouri-Attia N, Fahey AG, Browne J, Forde N, et al. (2013) Characterization of the Th Profile of the Bovine Endometrium during the Oestrous Cycle and Early Pregnancy. PLoS ONE 8(10): e75571. doi:10.1371/journal.pone.0075571
