# Supplementary material for: Characterization of the Th Profile of the Bovine Endometrium during the Oestrous Cycle and Early Pregnancy
Source: PLoS One. 2013 Oct 25;8(10):e75571. doi: 10.1371/journal.pone.0075571 (PMC3808391; doi:10.1371/journal.pone.0075571)
Supplement: File S1 — Trial Protocol. (DOCX) [file pone.0075571.s001.docx]

**Table S1: Primer sequences for QPCR**

| **Gene symbol** | **Gene name** | **Bos taurus gene ID** | **Primer** | **Sequence** | **Fragment size (bp)** |
| --- | --- | --- | --- | --- | --- |
| *BOLA-I*  *(MHC-I)* | Bovine Class I Major Histocompatability Complex | 782684 | Bov7-11.F | AGACGCGAATCTCCAAGGAA | 62 |
|  |  |  | Bov7-11.R | GCCGCGCAGGTGTT |  |
| *Foxp3* | Forkhead box P3 | 506053 | Foxp3.F | ATGCCCAACCCAAGGCCAGC | 123 |
|  |  |  | Foxp3.R | TGGGCTCTTGGTGCCCAGCT |  |
| *IFNA* | Interferon alpha | 515951 | IFN-Alpha.F | CTCCATGAGGTGACCCAGCAC | 121 |
|  |  |  | IFN-Alpha.R | GCAGGTCAGTGAGCTGCTGA |  |
| *IFNG* | Interferon gamma | 281237 | IFN gamma.F | TCAAATTCCGGTGGATGATCTGC | 150 |
|  |  |  | IFN gamma.R | GACCATTACGTTGATGCTCTCCG |  |
| *IL1A* | Interleukine 1 alpha | 281250 | IL1alpha.F | TTCGAGATATGTCAGGTCCATACC | 116 |
|  |  |  | IL1alpha.R | AGTCACAGGAAGCTGAGAATCC |  |
| *IL1B* | Interleukine 1 beta | 281251 | IL-1 beta.F | GCTTCAGGCAGGTGGTGTCGG | 101 |
|  |  |  | IL-1 beta.R | TGAAAGGATGCTCCTCAGGTCATCA |  |
| *IL2* | Interleukine 2 | 280822 | IL-2.F | AGAAGTGAAGTCATTGCTGCTGG | 121 |
|  |  |  | IL-2.R | TGTAGCGTTAACCTTGGGCG |  |
| *IL6* | Interleukine 6 | 280826 | IL- 6.F | ATCAGAACACTGATCCAGATCC | 145 |
|  |  |  | IL- 6.R | CAAGGTTTCTCAGGATGAGG |  |
| *IL8* | Interleukine 8 | 280828 | IL-8.F | GAAGAGAGCTGAGAAGCAAGATCC | 142 |
|  |  |  | IL-8.R | ACCCACACAGAACATGAGGC |  |
| *IL10* | Interleukine 10 | 281246 | IL-10.F | TGAAGGACCAACTGCACAGC | 121 |
|  |  |  | IL-10.R | TGTGGCATCACCTCTTCCAG |  |
| *IL11* | Interleukine 11 | 618708 | IL-11.F | GGGCCGCTGACCCGTAT | 121 |
|  |  |  | IL-11.R | GCAGACCCAGGTGTCGTTCA |  |
| *IL12A* | Interleukine 12 alpha | 281856 | IL12- alpha.F | AGGCCATGAATGCAAAGCTT | 121 |
|  |  |  | IL12- alpha.R | TGTGGCACAGTCTCACTGTCG |  |
| *LIF* | Leukemia inhibitory factor | 280840 | LIF.F | CCAGCTGGGACAACTCAACA | 121 |
|  |  |  | LIF.R | CGGGAAGTCAGTCACGTTGG |  |
| *PPIA* | Peptidylprolyl isomerase A | 281418 | PPIA-F | CATACAGGTCCTGGCATC | 108 |
|  |  |  | PPIA-R | CACGTGCTTGCCATCCAA |  |
| *ACTB* | Actin beta | 14186 | ACTB.F | CAGCAGATGTGGATCAGCAAGC | 91 |
|  |  |  | ACTB.R | AACGCAGCTAACAGTCCGCC |  |
| *RPL19* | Ribosomal protein L19 | 55433 | RPL19.F | CCCCAATGAGACCAATGAAATC | 73 |
|  |  |  | RPL19.R | CAGCCCATCTTTGATCAGCTT |  |

**Table S2: Fold change in candidate gene mRNA expression during the oestrous cycle^$^**

| **Gene ID** | **day 5vs day 7** | **day 5vs13** | **day 5 vs 16** | **day 7vs 13** | **day 7vs16** | **day 13vs16** |
| --- | --- | --- | --- | --- | --- | --- |
| *Bov 7/11* | 7.44 | 9.21 | 33.41 | 1.24 | 4.49 | 3.63 |
| *FOXP3* | 1.36 | 1.28 | 1.78 | -1.06 | 1.31 | 1.39 |
| *IFNA* | 3.15** | 2.67** | 1.35 | -1.18 | -2.33* | -1.97 |
| *IFNG* | -2.12 | 2.18 | 1.50 | 4.63*** | 3.19* | -1.45 |
| *LIF* | 1.78* | 3.20*** | 3.07*** | 1.79* | 1.72 | -1.04 |
| *IL1A* | 1.19 | 1.27 | 1.43 | 1.06 | 1.20 | 1.13 |
| *IL1B* | 1.51 | -1.25 | -1.96 | -1.89 | -2.95*** | 0.64 |
| *IL2* | 1.44 | 2.25 | 1.34 | 1.56 | -1.07 | -1.67 |
| *IL6* | -2.41 | -3.18 | -1.73 | -1.32 | 1.39 | 1.83 |
| *IL8* | -2.74 | -1.22 | 1.48 | 2.26 | 4.06** | 1.80 |
| *IL10* | -1.04 | 2.38 | 2.36 | 2.48 | 2.45 | -1.01 |
| *IL11* | 2.08 | 2.11 | 1.17 | 1.02 | -1.78 | -1.81 |
| *IL12A* | -1.82 | -1.19 | 1.24 | 1.53 | 2.26 | 1.47 |

*= p<0.05, **= p<0.01: ***= p<0.001 in all tissues. Negative values indicate fold change down regulation. ^$^Values generated from geometric least squared means

**Table S3: Fold change in candidate gene mRNA expression during preimplantation pregnancy^$^**

| **Gene ID** | **day 5vs day 7** | **day 5vs13** | **day 5 vs 16** | **day 7vs 13** | **day 7vs16** | **day 13vs16** |
| --- | --- | --- | --- | --- | --- | --- |
| *Bov 7/11* | 1.48 | 2.89 | 3.10 | -1.95 | -2.10 | -1.07 |
| *FOXP3* | 1.64 | 1.00 | -1.13 | -1.64 | -1.84 | -1.12 |
| *IFNA* | 1.76 | 1.28 | -1.22 | -1.38 | -2.16 | -1.57 |
| *IFNG* | 2.00 | 1.68 | 1.78 | -1.19 | -1.12 | 1.06 |
| *LIF* | 1.31 | 2.64*** | 1.94** | 2.01*** | 1.48 | -1.36 |
| *IL1A* | 1.02 | 1.15 | 1.47 | 1.13 | 1.44 | 1.27 |
| *IL1B* | 1.10 | -1.88 | -2.74** | -2.07* | -3.03** | -1.46 |
| *IL2* | 1.12 | 1.01 | 1.23 | -1.11 | 1.09 | 1.21 |
| *IL6* | 1.02 | -1.47 | 1.37 | -1.49 | 1.34 | 2.00 |
| *IL8* | -1.85 | 1.01 | 1.47 | 1.86 | 2.71 | 1.46 |
| *IL10* | 3.04* | 4.23*** | 3.54** | 1.39 | 1.16 | -1.19 |
| *IL11* | 1.20 | -1.24 | -2.78** | -1.49 | -3.34*** | -2.24* |
| *IL12A* | 1.56 | 1.47 | 2.02** | -1.06 | 1.29*** | 1.37* |

^$^Values generated from geometric least squared means. Negative values indicate fold change down regulation.*= p <0.05, **= p<0.01: ***= p<0.001 in all tissues.

Table S4: Summary of Immune Factor correlations in transcript expression*

| **Gene Name** | **Day of cycle /pregnancy** | **Status** | |
| --- | --- | --- | --- |
|  |  | **Cyclic** | **Pregnant** |
| *IFNA* |  |  |  |
|  | Day 5 | IL11, TGFB, NC2 |  |
|  | Day 7 | IL10, IL11, IL15, FOXP3, MCP1, PTX3 |  |
|  | Day 13 |  | IL10, IL18, IL12A, LIF |
|  | Day 16 |  | IL1A, IL1B, IL10, PTX3, RSAD2, NC2 |
| *IL1B* |  |  |  |
|  | Day 5 |  |  |
|  | Day 7 | IL12A, IL15, FOXP3 | IL15, CSF1, MCP2 |
|  | Day 13 |  | IL1A, IL6, IL11, IL12A, IL15, CSF1, ISG15, LIF, MCP2, TNFA, NC3 |
|  | Day 16 | IL1A, IL6, IL8, LIF, MCP1, MCP2 | IFNA, LIF, TNFA, PTX3 |
| *IL8* |  |  |  |
|  | Day 5 | IL1A, IL6, IL12A, MCP1, MCP2, TNFA |  |
|  | Day 7 |  |  |
|  | Day 13 | IL1B, NC3 |  |
|  | Day 16 | IL1B, IL6, ISG15, MCP1, MCP2, LIF |  |
| *IL10* |  |  |  |
|  | Day 5 |  | IL18,FOXP3, LIF, PTX3, NC3 |
|  | Day 7 |  | IL12A, IL15, LIF, TNFA |
|  | Day 13 |  | IFNA, FOXP3 |
|  | Day 16 |  | IFNA |
| *IL11* |  |  |  |
|  | Day 13 |  | IL1A, IL1B, IL6, IL12A, IL15, IL18, LIF, PTX3 |
| *IL12A* |  |  |  |
|  | Day 5 | IL6, IL8, IL11, IL15, MCP1, TGFB, JSP1 | IL1B, CSF1, MCP1, MCP2, ISG15, NC2 |
|  | Day 7 | IL1B, IL6, IL10, IIL15, IL18, CSF1, LIF, MCP2, TNFA, NC4 | IL1B, IL6, IL10, IIL15, IL18, CSF1, LIF, TNFA |
|  | Day 13 | RSAD2 | IL1A ,IL1B, IL6, IL11, IIL15, IL18, LIF |
|  | Day 16 | IL18 | IL10, IIL15, CSF1, LIF, TNFA |
| *LIF* |  |  |  |
|  | Day 5 | CSF1 | IL10 |
|  | Day 7 | IL6, IL12A, IL15, IL18, MCP1, TNFA | IL10, IL12A, IL15, IL18, MCP1, TNFA |
|  | Day 13 | IL15, ISG15, MCP1, MCP2, TGFB, NC4 | IL1A, IL1B, IL6, IL11, IL12A, IL15, IL18, IFNA,PTX3 |
|  | Day 16 | IL1A, IL1B, IL8, IL10, MCP1, MCP2 | IL1B, IL12A, IL15, IL18, TNFA |

*Pearson correlations were estimated between the expression profiles of a panel of 32 immune factors, including all genes analyzed for pregnant and cyclic cows at days 5, 7, 13, and 16 in the current study, to determine which factors were correlated. Although there was considerable interaction between factors, there was no clear influence of Th 1 type or Th 2 type cytokines. However, the influence of interferon-stimulated genes, *PTX3*, *RSAD2* and *ISG15* was primarily in effect on Days 13 and 16 of pregnancy, when their expression is highest [[31](#_ENREF_31)].
